# The Mildmay Cottage Hospital

**Published:** 1888-06-16

**Authors:** 


### THE MILDMAY COTTAGE HOSPITAL.

The report of the Mildmay Mission Hospital is well called "a light in a dark place." Labouring in Bethnal Green among people whose surroundings hardly permit either decency or health, they have succeeded in not only doing much for the cure of the diseases which poverty and unhealthy conditions of life generally make almost epidemic there, but in teaching many of their patients to lead a purer and higher life than they have ever thought of before. Though the work is hard and often discouraging, the workers have the great compensation of knowing that those whom they have benefited have profited not only during their time of illness, but throughout their whole lives, by the lesson they have then learnt.

